# Two-Dimensional High-Performance Liquid Chromatography as a Powerful Tool for Bioanalysis: The Paradigm of Antibiotics

**DOI:** 10.3390/molecules28135056

**Published:** 2023-06-28

**Authors:** Christina Papatheocharidou, Victoria Samanidou

**Affiliations:** Laboratory of Analytical Chemistry, School of Chemistry, Aristotle University of Thessaloniki, GR-54124 Thessaloniki, Greece; papatheocharidouchristina@gmail.com

**Keywords:** two-dimensional liquid chromatography, modes, instrumentation, antibiotics

## Abstract

The technique of two-dimensional high-performance liquid chromatography has managed to gain the recognition it deserves thanks to the advantages of satisfactory separations it can offer compared to simple one-dimensional. This review presents in detail key features of the technique, modes of operation, and concepts that ensure its optimal application and consequently the best possible separation of even the most complex samples. Publications focusing on the separation of antibiotics and their respective impurities are also presented, providing information concerning the analytical characteristics of the technique related to the arrangement of the instrument and the chromatographic conditions.

## 1. Introduction

The innovative idea of applying multidimensional techniques to challenging complex matrices such as blood, urine, biological cells, and environmental or forensic samples emerged due to the inability of simple methods to achieve high resolution in a short time [1]. The first application of two-dimensional chromatography was proposed by Consden, Gordon, and Martin when they used paper chromatography to separate 22 protein amino acids, within 120 h [2]. Then, Kirchner implemented thin-layer chromatography in 2D mode, turning it directly into a promising analytical separation tool. These applications were followed by the evolution of two-dimensional gas and liquid chromatography, the very primary separation techniques of recent years.

Two-dimensional liquid chromatography has deservedly won a dominant position in the family of LC techniques [3]. Even though liquid chromatography has great flexibility, regarding the different forms in which it has prevailed and the variety of samples it can analyze, it is unable to provide high resolving power in a short period of time. It is not able to deal with two types of mixtures: those consisting of thousands of analytes such as biological samples and those consisting of closely related compounds which cannot be separated by their physicochemical properties, such as enantiomers [3]. 

In fact, peaks of one-dimensional chromatograms overlap either with other compounds of related samples or with matrix components, although selectivity between different analyzers can be improved. There is not enough room to separate similar compounds, and max capacity is not sufficient for complex samples [1]. As a rule, a simple liquid chromatography system analyzes samples of 20 components in 2 h, and the time increases in proportion to the compounds. In addition, the incompatibility between the LC techniques and mass spectroscopy, which leads to the ion suppression phenomenon due to co-eluting compounds, is another issue that discourages LC application. This is particularly the case in the domain of pharmaceutical analysis, where the detection of impurities is mandatory [4]. Therefore, multidimensional separations can deal with complex and difficult mixtures if they are carried out by qualified operators [5].

In 2D-LC, two different separation modes are exploited to increase the maximum peak capacity and achieve the optimal separation efficiency. The first dimension separates the sample based on one set of physicochemical properties, related to molecular size and polarity, while the co-eluted compounds are subsequently transferred to the second dimension for a further separation [6]. To achieve this goal, the total analysis time is clearly increased compared to conventional liquid chromatography. The procedure of method development, the assembly of two different stationary phases, and the data processing stage are extremely time-consuming [7]. It should be mentioned that the 1D separation time influences the total analysis time, as 2D separations must be shorter [8].

## 2. General Aspects of 2D-LC

### 2.1. Historical Data

Two-dimensional separations are not a recent invention. The first mention of this type of chromatography was made in 1941 by Martin and Synge. They argued that two-dimensional separation of liquid samples, known as partition chromatography, depends on two factors: the rate at which the liquid mobile phase flows and the square of the diameter of the particles fixed in the separation column. After this paper, Dent and his colleagues used 2D paper for the isolation and separation of 19 amino acids from potatoes. The results of various subsequent attempts raised several questions in the scientific community, which was unable to provide a conclusive explanation of the divisions until 1967, when scientists discovered high-pressure liquid chromatography (HPLC), providing documented answers to key questions. In 1978, Erni and Frei conducted a very important experiment in which they tried to combine a gel permeation stationary phase with a reverse phase one, separating sienna glycoside [9]. In 1990, Bushey and Jorgenson applied for the first time the functionally complete technique of two-dimensional liquid chromatography. They separated 14 components contained in a protein mixture, combining size-exclusion and ion-exchange chromatography. The applications that followed were based on homemade heart-cutting mode for most samples, except for proteomics. In proteomics, scientists attempted to apply the comprehensive technique. The first decade of 2000 was followed by papers that highlighted some weaknesses of the technique, such as undersampling. Others highlighted features that upgrade the analysis in terms of both accuracy and time. Among the properties investigated were that of maximum capacity, the application of gradient elution to the second column, and the statistical processing of data. 

### 2.2. Modes of 2D-LC

**Figure 1:**
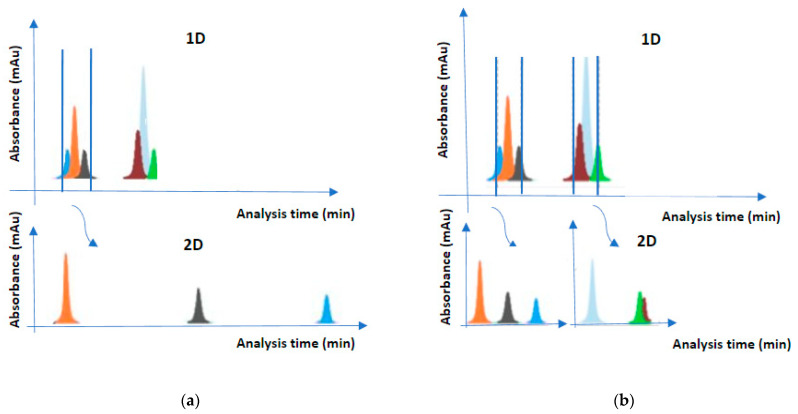
Heart-cutting mode (**a**) and multiple heart-cutting mode (**b**).

The heart-cutting mode is a simple and easy application of 2D-LC, which provides targeted analysis in a complex matrix (e.g., proteins in blood serum). The analytical process involves transferring one or even a small number of 1D fractions to the second column for further separation [3]. By focusing on the compounds of interest, selectivity and sensitivity are enhanced, thus improving detection limits. The operating cost is relatively low as it does not require specialized instruments compared to comprehensive mode. This setup increases 2D sampling time from 1D runtime. Consequently, the separation power of the total chromatograph increases, and higher separation efficiency is achieved (Figure 1). One disadvantage is the possible loss of important information due to selective transfer of compounds. The heart-cutting mode is very useful for identifying peaks, a very important tool for forensics [1]. Conventionally it is denoted as LC–LC.

The multiple heart-cutting mode is an intermediate mode between comprehensive and heart-cutting mode which provides broader flexibility to the simple heart-cutting mode. More specifically, this mode focuses on multiple peaks of interest which are transferred sequentially to the 2D column for further separation (the total separation process lasts a few seconds or a minute). The looping systems are connected to specific valves to store and purify the 1D eluents before their reinjection. When only the 2D column is ready for a separation, the sampling loop will permit the transfer among columns [3]. The multiple heart-cutting mode is the preferable method for the nonvolatile mobile phases with the ion source in mass spectroscopy [10]. However multiple heart-cutting increases complexity [11]. Conventionally, it is denoted as mLC–LC.

**Figure 2:**
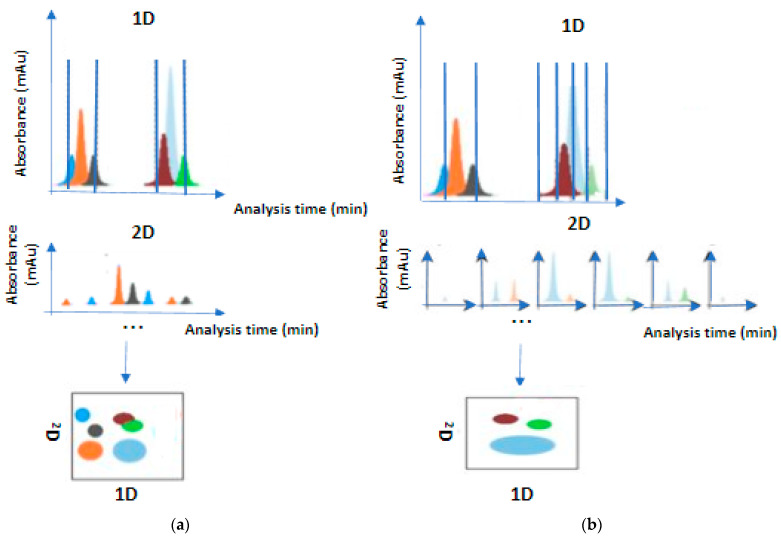
Comprehensive mode (**a**) and selective comprehensive mode (**b**).

The comprehensive mode was proposed after the heart-cutting mode’s innovation [12]. It expanded the field of applications and offered a total analysis of the sample, especially for the compounds consisting of thousands of metabolites or co-eluted 1D fractions. Every compound which is spotted in the sample is compulsorily passed through the whole analysis system [4]. The 1D eluents are temporally stored in loops (the most common form) [13] until the 2D column performs further separations (Figure 2). The 2D separations must be faster than those of 1D. The primary goal of a complex fraction is to obtain as much information as possible about the sample by extracting a two-dimensional chromatogram [3]. In simple case studies, the goal of the technique is the successful separation of all components. Sampling time is frequent to avoid remixing fractions that have been successfully separated in the first dimension. Thus, this setup requires short columns with a significant rate difference between columns, where the 2D rate must be higher. This technique is particularly widespread in the fields of omics technology [14]. It is very useful for composite samples consisting of nonvolatile samples. It involves a more extensive separation process, resulting in a higher overall analysis time [1]. It requires specialized operation and application of chemometric methods, increasing the operating cost [15]. A significant advantage of the comprehensive mode is the ability to be combined with mass spectroscopy, eliminating the problems arising from conventional LC–MS. LC × LC/MS is a three-dimensional separation system which prevents matrix effects, offering quantitative analysis and identification of even unknown compounds [16]. Conventionally, it is denoted as LC × LC.

A basic prerequisite for the application of the comprehensive technique is that the separation of 2D fractions and the sampling time of the next 1D eluent should be carried out simultaneously. Such limitations complicate the technique’s implementation compared to one-off heart cutting. Time constraints become sampling limitations, since the volume of sample that can be hosted in loop is specific and limited [3]. Under perfect conditions, each 1D fraction should be transferred to the second dimension in three or four consecutive fractions. The maximum allowable pressure in both dimensions determines the time of a full LC × LC analysis. The selective comprehensive mode is a hybrid one that excellently combines the principles of the heart and comprehensive techniques. It can perform comprehensive separations, while still focusing on specific analytes. This specific feature has a double-positive impact: it restricts the problem of undersampling peaks in the first dimension, and it shortens the analysis time without taking under consideration the empty content of 1D separation. The selective comprehensive mode is used for quantitative purposes due to the ability to provide high resolution [17,18]. It cannot be compared with the multiple heart-cutting setup, although similar instrument hardware can be used [3]. Conventionally, it is denoted as sLC × LC.

Scientists developed the hybrid multiple heart-cutting and selective comprehensive mode to overcome the difficulties that arise when applying traditional methods. This approach can perform the basic concepts of a conventional 2D-LC method, while offering some extra advantages. However, its application is not always possible. Table 1 summarizes the benefits and drawbacks of two-dimensional liquid chromatography.

### 2.3. Classification of Two-Dimensional Techniques Based on Temporal Transfer of Fractions

In offline mode, fractions from the first column are processed before being injected into the second column. The storage of fractions in vials for reinjection is a factor contributing to contamination or sample loss. It can be performed with an LC system using the same instrument if there are complementary mechanisms among the two dimensions. The first dimension operates continuously while the analysis time of the second column does not affect the total analysis time [3,12]. This is a significant benefit over the other modes as it can be very convenient for a variety of applications. However, the offline mode is time-consuming and cannot be performed in an automated manner [4].

In online mode, the technique can be fully automated until the data processing stage and is much faster than offline mode. The two columns are combined via a specific interface (a two-position switching valve of eight or six ports equipped with two storage loops of the same volume), responsible for the direct/continuous transfer of eluents between columns. An important advantage of online modes is full control of the sample, as well as the fact that there is no possibility of loss. Nevertheless, the implementation of online mode requires complex settings for the utilization of data, and it offers lower peak capacity than offline mode [3,12].

The stop-and-go approach is a compromise in terms of time and power of analysis compared to the previous approaches. The analytical procedures among the two dimensions are carried out alternately. When a specific volume of mobile phase has passed through the 1D column, the flow of the 1D mobile phase is temporarily stopped, allowing the fractions to be retained on the 1D column. Then, the 2D column is ready to start the separation without using sampling loops [3].

## 3. Orthogonality

The word orthogonal is a combination of the two Greek acronyms “ortho” and “gon”, meaning right and angled, respectively. In 2D-LC, orthogonality refers to the degree of independence achieved between the two separation dimensions [19]. The dimensions are uncorrelated and unable to influence each other. The orthogonal characteristic of 2D-LC improves the analytical procedure, enhancing peak capacity and achieving the maximum separation efficiency. Any attempt to apply these systems can be very challenging as scientists must choose carefully among the variety of different columns, which is also very important for accurate results [20]. The appropriate combinations of stationary phases are selected by considering the physicochemical properties of the relevant sample. The possible combinations that can be achieved are RPLC × RPLC, HILIC × RPLC, IEC × RPLC, SEC × RPLC, and NPLC × RPLC [3].

**Figure 3:**
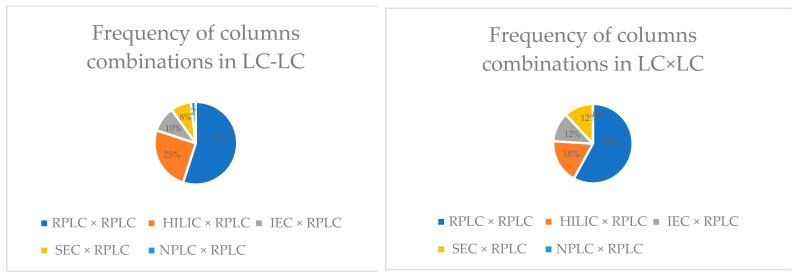
Frequency of column combinations in LC–LC and LC × LC analyses [1].

The final choice of separation mechanisms depends on the analytical purpose, the types of analytes, and the matrix. The frequency of column combinations is illustrated in Figure 3.

Some of the above combinations are theoretically possible but practically impossible [3]. When the solvents of mobile phases are immiscible, then the relative mobile phases are incompatible. Generally, mobile phases with different viscosities result in unstable flows, due to the inability of the stationary phases to hold the compounds. A typical example of solvent incompatibility is the normal/reversed phase combination, which has a limited number of practical applications, mostly in offline modes. On the other hand, there are other case studies in which the limited peak capacity is compensated for by the orthogonality achieved. For example, the implementation of ion-exchange or size-exclusion chromatography with reversed-phase chromatography. These combinations are widely used in trace analysis and chiral analysis. Essentially, IEX and SEC columns are used for impurity removal, while RP columns are used for component separation. However, column equilibration requires substantial time for analysis, and the mobile phase is not compatible with MS detectors, a very important tool in analytical separations [4]. The application of reversed-phase chromatography in both dimensions is most suitable in the two-dimensional field. The high separation efficiency, the generation of maximum peak capacity, the commercial availability of RP stationary phases, and the compatibility of the relative solvents with the MS detector are some of the attributes that enhance RP–RP applications [21]. However, the double RPLC combination lacks orthogonality compared to the above combinations [22]. A very promising combination is HILIC–RP, as the mobile phases used in HILIC are similar to those used in RP, resulting in orthogonality [4].

The simplest way to combine different separation mechanisms is passive modulation, which is based on an interface valve with two identical storage loops. When the 1D eluent is sampled by the first loop, the second one transfers the previous eluent into the second column. However, there are cases in which solvent incompatibility broadens or distorts peaks and leads to difficult quantitation [1]. Scientists have tried addressing this difficulty by making modifications to the modulation process. Some cases are distinguished below.

In active-solvent modulation, the 1D eluents are prediluted in the second mobile phase by a weak solvent before they proceed to the 2D column. There is an eight-port valve with four positions, two of which are functionally identical to those of passive modulation. The content of the second pump is separated by the other two positions and travels through the bypass capillary. This part of the flow joins the stream of fluid coming from the sampling loop before the sample returns in the second column. In this way, the separated flow portion of the second column acts as a diluent [23].

In stationary-phase-assisted modulation (SPAM), the sampling loops are replaced by small-volume trapping columns with which increase the sample’s concentration while reducing problems that occur during injection. These columns can be combined mostly with a ten-port valve. Even though the sensitivity of the method can be highly increased, this modulation is not so simple to implement. The stationary phase is chosen according to the physicochemical properties of the sample and the types of mobile phases in both dimensions, choices that can be very challenging. In addition, it is considered the most used but unstable modulation due to the possible premature elution of the analytes. This technique is suitable for multiple heart cutting if the traps are identical [24].

Vacuum-evaporation modulation includes the evaporation of 1D eluents to remove the solvent of the first dimension [1]. During switching, the mobile phase of the second column redissolves the specified compounds and introduces them into the second column for further separation. This setup is often used in noncomprehensive modes, and, although its advantages have been demonstrated, there are still some questions. The first refers to the volatility of the evaporated solvent, the second concerns compounds that cannot be easily dissolved, and the last focuses on the potential loss of specific analytes [25].

## 4. Peak Capacity

According to Giddings, peak capacity is the maximum number of equal peaks that can be placed between an early and a late peak in a chromatogram [26]. In other words, it is a term that describes the ability of the analytical technique to separate multiple peaks in a single run. The chief advantage of 2D-LC is the second dimension’s ability to increase peak capacity without increasing the overall analysis time. The orthogonality achieved between two dimensions increases separation’s efficiency, resulting in maximum peak capacity. The relative calculation is as follows: peak capacity = number of peaks obtained from the 1D × number of compounds obtained from the 2D [27]. It is well known that the increase in resolution of each dimension contributes to the overall calculation of peak capacity. However, several parameters determine the peak capacity of a 2D-LC application, such as the number of theoretical plates (N) and the separation selectivity α. 

To determine these parameters, despite the easy application and the high speed of isocratic elution, despite the instrument and the columns not needing re-equilibration after each run, gradient elution is the most applicable method [28,29]. The peaks of gradient chromatography are sharper and narrower with equal peak widths compared to the wider peaks of isocratic elution. This attribute provides better quantifications, especially for the late-eluting fractions. In gradient elution, the constant change in the characteristic retention factor broadens the range of analytes that can be separated. Its greatest advantage is that it avoids dilution during the outflow from the first column and the introduction of the sample to the top of the second column, which occurs during isocratic elution [30].

## 5. Undersampling

The problem of undersampling refers to a small set of 1D compounds transferred to the second column, degrading the quality of the results. The compounds that are not transferred are mixed in the sampling loop of 1D, with their immediate subsequent loss. The key parameter involved in the exemplary condition is the sampling frequency of the first column (when carried out late), as well as the inability to implement all necessary processes to ensure the full coverage of peak capacity [3]. The loss of specific fractions during transfer from one column to another is inevitable, but not undetectable. Therefore, the product rule of peak capacity is adjusted to add the correction factor Cf: peak capacity = number of peaks obtained from the 1D/Cf × number of compounds obtained from the 2D [19]. Consequently, the Giddings definition of maximum capacity cannot be fully applied, taking into consideration the losses that occur. 

## 6. 2D-LC Instrumentation

The individual parts of a 2D-LC instrument are significantly different from those of a one-dimensional liquid chromatography system. Both components and data processing software have made significant progress, transforming homemade instrumentations into advanced systems that perform analyses depending on their assembly. The most important parts of the instrumentation are outlined below.

Pumping system: The pumping system is determined by two main attributes. The first one is the gradient delay volume, i.e., the solvent volume which is delivered by the pump before the 2D column accepts the solvent of the mobile phase. The second one is the flush-out volume, which refers to the use of an organic solvent that will wash the column after every analysis. Modern 2D-LC comprehensive separations have pumps with a volume of 100 mL, and they are capable of gradient elution in a short time. If the system does not retain such requirements, undersampling can occur. To improve the capabilities of pumps, the use of parallel identical columns is recommended in terms of efficiency and retention mechanisms. The systems have totally been improved compared to past pumps with a capacity of 1 mL. In heart cutting, the volume of the pumping system is not investigated, as the execution time of each 2D separation is sufficient [6]. 

Columns: The two different columns have different dimensions and stationary phases. The final choice is based on the analyte to be separated [12].

**Figure 4:**
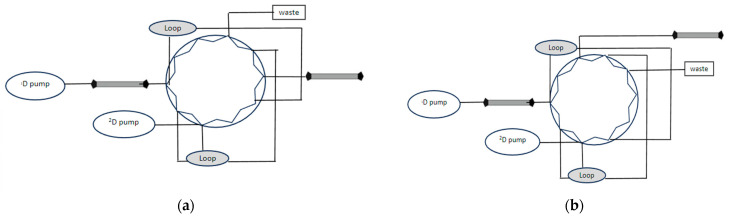
(**a**) Symmetrical arrangement. (**b**) Asymmetrical arrangement.

Interface valve: The interface valve is located between the separation columns, and it is responsible for the transformation of 1D eluent to 2D. It affects the performance of the separation column, the detection of the identified components, and therefore, the quantification of the results. Due to its usefulness, one- or two-position valves with six (only applicable for heart-cutting mode), eight, 10, or 12 loops (applicable in comprehensive mode), or even two-port valves with six loops have been invented. Every valve consists of a valve motor, stator, and rotor [31]. The most common type among all practical combinations is a two-position valve connected with two columns, while the most common model is asymmetrical instead of symmetrical, as shown in Figure 4 [31]. The results of Van der Horst’s research, however, overturned the current data when he reported that the direction of samples within the sampling loops has a significant effect on the retention time of the determined components. Therefore, the use of asymmetric valves should be avoided in cases where the first column is characterized by a very slow speed.

Detectors for data acquisition and analysis: The detector system is an integral part of the second dimension, while it is often optional for the first one. The most common detectors are UV/Vis detectors, mass spectrometers, diode array detectors (DAD), photo array detectors (PDA), and fluorescence detectors [32]. However, no one detector can be effective without the appropriate system responsible for converting the generated signal into chemical information. The processes that take place refer to the quantification and identification of chromatography peaks [3]. 

**Figure 5:**
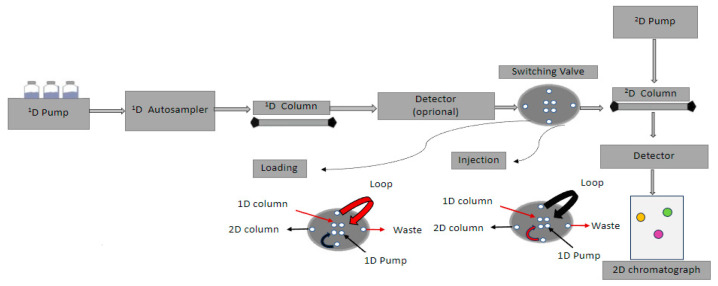
A typical 2D-LC system.

The visual representation of 2D data includes counter plots where the *x-* and *y*-axes represent the retention times of the two dimensions. The color of the plots is proportional to the intensity of the vertices. Therefore, the extracted chromatographic information develops an absorption area of specific values based on retention times, creating a three-dimensional data representation using special software (Fortner Transform) [33]. The quantification of a 2D chromatograph is a very challenging procedure, especially for the analytes of biological matrices. The utilized chemometric tools (e.g., ChromoSquare from Shimadzu), which include statistical models, have a fairly high cost and cannot be applied to all fields. To address these limitations, new approaches, which do not require specialized software, have been proposed. The strategies are based on the concept “region of interest “(ROI) and the design of multivariate calibration curves [34]. A typical 2D-LC system is illustrated in Figure 5.

## 7. Automation of 2D-LC

The application of online 2D-LC was the first recorded type of automation that upgraded the overall analysis in terms of time and efficiency [8]. However, the scientific community is still trying to shorten the analytical procedure by focusing on the instrument’s operation and the method’s optimization [1]. Some relative examples are discussed. Jintgal et al. proposed the detection and qualification of monoclonal antibody therapeutics at pg/mL levels with clearly increased sensitivity compared to the conventional LC–MS. The automation referred to the design of 2D-LC instrumentation, including the dilution of a specific analyte using an organic solvent, while two RPLC columns with different pH values were used. The separation time (1D and 2D) was only about 20 min [35]. Another study described the automation of the online selective comprehensive and multiple heart-cutting mode for the quantification of different forms of amino acids, by derivatizing the analytes before their injection into the separation columns, in just 45 min [36].

## 8. The Application of 2D-LC in Antibiotics Analysis

Antibiotics are chemotherapeutic drugs that destroy bacterial infections through preventive or therapeutic actions. They are low-molecular-weight compounds with different physicochemical characteristics. Based on their mechanism of action, antibiotics are used to treat a wide range of diseases such as pneumonia, syphilis, and septicemia. The bacterial cell wall consists of peptidoglycan, which is responsible for the basic functions of the bacterium. Hence, broad classes of antibiotics including beta-lactams, glycopeptides, cyclic peptides, and lipoglycopeptides act against the cell wall through peptidoglycan inhibition [37]. The consumption of antibiotics for a long time, in combination with the appearance of changes in the clinical picture of the patient, can lead to unpredictable concentrations of drugs in the human body. Their quantification has so far been highly focused on toxic antibiotics such as glycopeptides. However, therapeutic drug monitoring (TDM) involves quantitative testing of all possible classes, especially beta-lactams [38]. Interest is focused on the free fraction of pharmaceutical compounds that remains active, i.e., its free concentration in plasma, tissues, etc.

### Study Cases

Here, we discuss papers that describe the identification or quantification of different antibiotics and their impurities using 2D-LC. Processes such as polymerization, isomerization, degradation reactions, or even the storage of antibiotics in packages result in impurities which are implicated in causing allergies.

The first group of papers focused on resolving the incompatibility between the nonvolatile mobile phase and mass spectroscopy. The use of a normal, reversed, hydrophilic, or ion-exchange stationary phase in the first dimension is combined with a nonvolatile mobile phase for better separation. However, it is well known that most LC methods use mass spectroscopy detectors with electrospray ionization, showing a preference for volatile mobile phases. Their combination is challenging in 2D-LC application. Multiple heart-cutting mode in coordination with the appearance of trap-free columns provides a fast transportation of 1D eluents to the second column. Cefpiramide and eight impurities were determined using trap-free 2D-LC, allowing the detection of even low concentrations through the injection of a large sample volume. The results showed seven new impurities [39]. The identification of impurities in cefonicid sodium followed the same method, resulting in the identification of seven unknown degradation products of the main compound [40]. The application of multiple heart-cutting mode in combination with demineralization offered a fast and convenient technique. The simultaneous successful 2D-LC separation of polymerized impurities in cephalosporins (cefodizime, cefmenoxime, and cefonicid) followed the same method [41].

The second group of studies applied the 2D-LC technique to provide accurate results. A heart-cutting method was used for the qualitative and quantitative determination of vancomycin. Through two-dimensional separation combined with a third intermediate column, it was possible to inject a large sample volume (200 μL) and reduce the total analysis time to 4 min in contrast with a 7.5 min UPLC–MS/MS method. The 1D column was used as a cleaner agent while the 2D column was used as a separator. Therefore, any pre-treatment process such as SPE or LLE was not necessary. The lower limit of detection was 0.20 μg/mL [42]. Using 2D-LC, scientists tried to study the active ingredient of meropenem and quantify the ring-opened meropenem and meropenem dimer, known as A and B, with LODs of 0.07 μg·mL^−1^ and 0.22 μg·mL^−1^, respectively, and LOQs of 0.25 μg·mL^−1^ and 0.74 μg·mL^−1^, respectively. 2D-LC was also used to identify three new degradation impurities, and the results were evaluated using ESI-MS/MS data [43]. Another paper described the quantification of amoxicillin, benzylpenicillin, benzylpenicillin, flucloxacillin, and piperacillin, as well as the beta-lactamase inhibitors clavulanic acid and clindamycin, macrolide antibiotics, and tazobactam clindamycin [44]. Scientists studied plasma and tissue samples from surgical rehabilitation operations. All matrices were submitted to precipitation of their protein content, applying online extraction using the 1D column and separation using the 2D column. In plasma and plasma ultrafiltrate, the inaccuracy and imprecision for any analyte remained below 15%. In tissue, the accuracy and precision varied up to 16% and 20%, respectively, when various tissues were analyzed after water calibration. In addition, studies quantified oxacillin, cloxacillin, α-amoxicillin, and linezolid in human plasma using the same method. The innovation of this method was the application of 5-sulfosalicylic acid dihydrate (SSA) solution as a precipitation agent of substrate proteins [45]. Amoxicillin’s impurities were also studied separately. To separate high-molecular-weight impurities in amoxicillin, a heart-cutting mode was used, providing an eco-friendly separation technique. Gel filtration chromatography was combined with reversed-phase LC [46]. In 2022, two different methods were developed to determine polymerized impurities in cefotaxime sodium and cefepime [47]: an HPSEC TSK gel column, which provided co-induced impurities with lower molecular weights than the identified compounds, and an RP–RP technique, which offered higher sensitivity and selectivity. For the separation of sulfonamides, beta-agonists, and (steroid) hormones in urine samples, a home-made device was initially developed, providing low limits of detection (LOD) ranging from 1 μg/L to 10 μg/L. A comprehensive 2D-LC was further used for more sensitive results, applying the pretreatment technique of 96-well plates for urine precipitation [48]. The experimental conditions, under which all the above applications were carried out, are described in Table 2.

## 9. Conclusions

Undoubtedly, 2D-LC has set new standards in the domain of analytical chemistry. The ability to combine two different separation mechanisms has improved the resolution and detection limits of complex and closely related compounds. In some cases, pretreatment techniques have been replaced by using appropriate columns to clear and separate the matrix. This is easily combined with mass spectroscopy as a valuable comparison tool that can solve various problems during method development. On the other hand, there are some drawbacks that discourage the method’s application, e.g., the acquisition of appropriate instrumentation and instrumental complexity. Moreover, the overall analysis time is increased in the case of multiple cuts. Nevertheless, 2D-LC will become one of the most applicable separations techniques as more and more scientists adjust their theoretical backgrounds with the practical implementation of 2D-LC modes.

## Figures and Tables

**Table 1:** Benefits and drawbacks of hybrid modes of two-dimensional liquid chromatography.

Advantages	Disadvantages
The ability to combine different separation mechanisms increases selectivity by focusing on different analyte properties.	The coordination of all parameters required for the multiple combinations increases system complexity.
The different columns are combined, leading to orthogonal systems that can achieve improved separation efficiency.	The system’s complexity increases the overall analysis time, a primary limitation for time-sensitive applications.
The introduction of new orthogonal systems increases the peak capacity and the resolution power of the analytes of interest.	The use of multiple loops, columns, and valves necessitates upgrading the chromatographic system, whereby new modifications must be implemented.
Scientists broaden the fields of application to even the most complicated matrices.	Scientists consume time in searching for sources and, therefore, developing methods.

**Table 2:** Experimental conditions for several antibiotics.

Antibiotic	Column Type	Mobile Phase	Program’s Elution	Detector	References
Cefpiramide and relative impurities	1D: Kromasil C8 analytical column (250 mm × 4.6 mm, 5 µm). 2D: Shimadzu Shim-pack GISS C18 analytical column (124 mm × 2.1 mm, 1.9 µm).	1D: (A) 30 mM phosphate/methanol (75:25, *v*/*v*) and (B) phosphate buffer (30 mM, pH 7.5)/methanol (50:50, *v*/*v*) / 2D: (A) ammonium formate solution (10 mM) and (B) methanol.	1D: gradient conditions: Time (min) B% 0 0 12 0 32 100 33 0 The mobile phase flow rate was 0.80 mL/min. 2D: gradient conditions: Time (min) B% 0 5 5 95 5.5–9 5 The mobile phase flow rate was 0.30 mL/min.	Ion trap/time-of- flight mass spectrometer (Shimadzu Corp., Kyoto, Japan), equipped with an electrospray ionization (ESI) source in positive and negative mode.	[39]
Polymerized impurities of cephalosporins: cefodizime, cefmenoxime, and cefonicid	1D: Xtimate SEC-120 analytical column (7.8 mm × 30 cm, 5 m). 2D: Method A: Shimadzu Shim-pack GISS C18 analytical column (50 mm × 2.1 mm, 1.9 m). Method B/C: ZORBAX SB-C18 analytical column (4.6 × 150 mm, 3.5 m).	1D: (A) 0.005 mol/L dibasic sodium phosphate solution and (Β) 0.005 mol/L sodium dihydrogen phosphate solution (61:39, *v*/*v*)] acetonitrile *v*/*v*)]. 2D: Method A: (A) ammonium formate solution (10 mM) and (B) acetonitrile. Method B: (A) acetic acid solution (0.1%, *v*/*v*) and (B) acetonitrile. Method C: (A) ammonium formate solution (10 mM) and (B) ammonium formate (8 mM) in [acetonitrile/water (4:1, *v*/*v*)] solution.	1D: gradient conditions: Time (min) B% 0 5 18 20 20 5 The mobile phase’s flow rate was 0.80 mL/min and the injection volume was 30 μL. 2D: gradient elution Method A: Time (min) B% 0 5 5 95 5.5–9 5 Method B: Time (min) B% 0 5 12 15 45 60 55 5 Method C: Time (min) B% 0 12 9 16 15 20 18 40 19 12 The mobile phase flow rate was 0.40 mL/min.	Ion trap/time-of- flight mass spectrometer (Shimadzu Corp., Kyoto, Japan), equipped with an electrospray ionization (ESI) source in positive and negative mode.	[41]
Impurities in cefonicid sodium	1D: GRACE Alltima C18 analytical column (250 mm × 4.6 mm, 5 μm)/2D: Shimadzu Shim-pack GISS C18 analytical column (50 mm × 2.1 mm, 1.9 μm).	1D: (A) 0.02 mol·L^−1^ ammonium dihydrogen phosphate solution in H_2_O with 40% aqueous ammonia solution) and (B) methanol. 2D: (A) 10 mmol·L^−1^ ammonium formate solution and (B) methanol.	1D gradient elution: Time (min) B% 0–10 16 10–30 60 41 16 The mobile phase flow rate was 0.80 mL/min. 2D gradient elution: Time (min) B% 0 5 5 95 5.5–9 5 The mobile phase flow rate was 0.30 mL·min^−1^.	Ion trap/time-of- flight mass spectrometer (Shimadzu Corp., Kyoto, Japan), equipped with an electrospray ionization (ESI) source in positive and negative mode.	[40]
Meropenem	1D: Shim-Pack CLC ODS (6 mm × 150 mm, 5 μm, Shimadzu, Kyoto, Japan)/2D: Shim-pack GISS C18 column (50 mm × 2.1 mm, 1.9 μm, Shimadzu, Kyoto, Japan) used at 40 °C.	1D: (A) 0.1% triethylamine/acetonitrile (96:4, *v*/*v*) and (B) 0.1% triethylamine/acetonitrile (70:30, *v*/*v*) / 2D: (A) 10 mM ammonium formate and (Β) methanol.	1D: Gradient elution Time (min) B% 0 0 18 10 55 60 56 0 The flow rate was 1.5 mL·min^−1^. 2D: Gradient elution mode: Time (min) B% 0 5 5 95 5.5–9 5 The flow rate was 0.30 mL min^−1^.	Ion trap/time-of- flight mass spectrometer (Shimadzu Corp., Kyoto, Japan), equipped with an electrospray ionization (ESI) source in positive and negative mode.	[43]
Amoxicillin	1D: Shim-pack GIS C18 (4.6 mm × 250 mm, 5 μm)/2D: Shim-pack GIS C18(4.6 mm × 150 mm, 5 μm).	1D: ammonium dihydrogen phosphate/tetrahydrofuran/methanol (730∶12.5∶300) / 2D: 1% formic acid aqueous solution (A)/acetonitrile (B).	Isocratic conditions: the flow rate was 1 mL/min.	UV detector at 254 nm.	[46]
Benzylpenicillin, Flucloxacillin, Amoxicillin Riperacillin, the beta-lactamase inhibitors Clavulanic acid and Clindamycin, Macrolide antibiotics, and Tazobactam clindamycin	1D: XBridge^®^ C8 Direct Connect HP column (10 µm particle size, 2.1 × 30 mm)/2D: Acquity UPLC^®^ BEH C18 (1.7 µm particle size, 2.1 × 100 mm i.d.; Waters) analytical column equipped with an Acquity UPLC^®^ BEH C18 VanGuard precolumn (1.7 µm particle size, 2.1 × 5 mm i.d.; Waters).	1D: 100% H_2_O with 0.1% formic acid/2D: (A) water containing 0.1% formic acid and (B) acetonitrile containing 0.1% formic acid.	1D: Isocratic elusion at a flow rate of 1.0 mL/min. 2D: Gradient elution Time (min) B% 0.0 10 1.8 10 4.5 45 5.0 100 6.0 100 6.1 10 The flow rate was 700 µL/min.	A triple-quadrupole mass spectrometer (TSQ ENDURA) (Thermo Scientific, Reinach, Switzerland), equipped with an electrospray ionization source in positive mode.	[44]
Vancomycin	1D: Reversed-phase Diamonsil C18(2) column (100 mm × 4.6 mm, 5 μm, China; C1). Middle column: a strong cation-exchange column (20 mm × 4.6 mm, 5 μm, ANAX, China; MC). 2D: Inertsil ODS-3 column (150 mm × 4.6 mm, 5 μm, GL Science Inc., Japan; C2).	1D: (A) 20 mmol/L ammonium acetate buffer and (Β) acetonitrile (88:12, *v*/*v*)/ 2D: (A) 50.0 mmol/L Ammonium acetate buffer (pH 5.0) and (B) acetonitrile (85:15, *v*/*v*), mobile phase C.	1D: isocratic elusion with a flow rate of (A) 1.2 mL/min, (Β) 1.6 mL/min, and (C) 1.5 mL/min.	UV detector at 282 nm.	[42]
Amoxicillin, cloxacillin, oxacillin, and linezolid	1D: Perfusion column (POROS R1/20, 20 m, 2.1 mm × 30 mm, Applied Biosystems, Darmstadt, Germany)/2D: Pentafluorophenyl (PFP) analytical column (Phenomenex Kinetex, 2.6 m, 2 mm × 50 mm, Aschaffenburg, Germany).	1D: (A) water + 0.1% formic acid, (B) MeOH + 0.1% formic acid, (C) water/10 mM ammonium formate, with formic acid, and (D) ACN + 0.1% formic acid.	Isocratic conditions: a flow rate of 4.0 mL/min over 0.70 min. Gradient conditions: Time (min) D% 0 10 0.75 10 3.20 98 3.80 10 3.81 10	A TQD triple-quadrupole mass spectrometer) equipped with an electrospray ionization source (Waters, St Quentin, France) in positive mode.	[45]
Sulfonamides, Beta-agonists, and (Steroid) hormones	Self-made: 1D: Waters HSS Cyano (1.8 µm, 1 × 150 mm), Waters BEH C18 (1.8 µm, 1 × 150 mm), Waters Phenyl (1.8 µm, 1 × 150 mm) and a Phenomenex Kinetix (2.6 µm, 1.0 × 150 mm) column. 2D: Waters Phenyl column (1.7 µm, 2.1 × 50 mm). Commercial LC × LC	1D: (A) water/acetonitrile (90:10) containing 0.1% formic acid and (Β) water/acetonitrile (10:90) containing 0.1% formic acid. 2D: (A) water/acetonitrile (90:10) containing 0.1% formic acid and (B) water/acetonitrile (10:90) containing 0.1% formic acid.	1D gradient conditions: Time (min) B% 0 80 27 80 28 0 2D gradient conditions: Time (min) B% 0 0 45 40 50 100	Ion trap/time-of- flight mass spectrometer (Waldbronn, Germany), was equipped with an electrospray ionization (ESI) source in positive mode.	[48]
The polymerized impurities in cefotaxime sodium and cefepime 2D HPSEC/RP-HPLC system 2D RP-HPLC/RP-HPLC system	1D: a TSK-gel G2000SWxl column (7.8 mm × 30 cm, 5 μm) from TOSOH Corporation (Tokyo, Japan). 2D: Agilent ZORBAX SB-C18 analytical column (4.6 mm × 150 mm, 3.5 μm) (Santa Clara, CA, USA). 1D: Kromasil (Nouryon, Bohus, Sweden) 100-5-C18 analytical column (4.6 mm × 250 mm, 5 μm). 2D: Shimadzu Shim-pack GISS C18 analytical column (50 mm × 2.1 mm, 1.9 μm)	1D: phosphate buffer of dibasic sodium phosphate solution/0.005 mol/L sodium dihydrogen phosphate solution, 61:39 (*v*/*v*), and acetonitrile at 95:5 (*v*/*v*). 2D: (A) 10 mM ammonium formate solution and (B) acetonitrile. 1D: For cefotaxime sodium injection, the mobile phases were 0.05 M disodium hydrogen phosphate solution (A) and methanol (B). For cefepime, the mobile phases were 0.05 M ammonium dihydrogen phosphate solution (A) and acetonitrile (B). 2D: (A) 10 mM ammonium formate solution and (B) acetonitrile.	1D: Isocratic conditions: a flow rate of 0.50 mL/min 2D: Gradient elution Time (min) B% 0–20 5 20–21 40 21–29 5 The flow rate of the mobile phase was 0.40 mL/min. 1D for cefotaxime, gradient elution: Time (min) B% 0–10 15 10–30 15 30–40 70 40–41 70 41–50 15 The flow rate was 0.8 mL/min and the injection volume was 20 μL. 1D for cefepime: the gradient program was set as follows: Time (min) B% 0 10 10 10 30 70 40 70 41–50 10 The flow rate was 1.00 mL/min. 2D, gradient conditions: Time (min) B% 0–5 5–95 5.5–5.5 5	1D: PDA detector in the range of 200–400 nm. 2D: UV detection wavelength of 254 nm. 1D: PDA detector in the range of 200–400 nm. 2D: Ion trap/time-of-flight mass spectrometer (Shimadzu Corp., Kyoto, Japan), equipped with an electrospray ionization (ESI) source in positive and negative mode.	[41]

## Data Availability

Not applicable.

## Sample Availability

Not available.
